# Macrophage MST1 protects against schistosomiasis-induced liver fibrosis by promoting the PPARγ-CD36 pathway and suppressing NF-κB signaling

**DOI:** 10.1371/journal.ppat.1012790

**Published:** 2024-12-19

**Authors:** Jianyang Li, Xinyuan Cai, Yan Yang, Yulin Mao, Lin Ding, Qian Xue, Xunhao Hu, Yan Huang, Cong Sui, Yuxia Zhang

**Affiliations:** 1 School of Basic Medical Sciences, Anhui Medical University, Hefei, Anhui Province, P.R. China; 2 The First Affiliated Hospital of Anhui medical University, Hefei, Anhui, China; 3 The First Clinical Medical College of Anhui Medical University, Hefei, China; 4 Inflammation and Immune Mediated Diseases Laboratory of Anhui, Hefei, China; University of Medicine & Dentistry New Jersey, UNITED STATES OF AMERICA

## Abstract

Schistosomiasis is characterized by egg-induced hepatic granulomas and subsequent fibrosis. Monocyte-derived macrophages play critical and plastic roles in the progression and regression of liver fibrosis, adopting different polarization phenotypes. Mammalian STE20-like protein kinase 1 (MST1), a serine/threonine kinase, has been established to act as a negative regulator of macrophage-associated inflammation. However, the specific role of MST1 in *Schistosoma*-induced liver fibrosis has not been fully understood. In this study, we demonstrate that macrophage MST1 functions as an inhibitor of inflammation and fibrosis following infection with *Schistosoma japonicum (S*. *japonicum)*. Mice with macrophages-specific *Mst1* knockout (termed *Mst1*^*△M/△M*^) mice developed exacerbated liver pathology, characterized by larger egg-induced granulomas, and increased fibrosis post infection. This was accompanied by enhanced production of proinflammatory cytokines (IL1B, IL6, IL23, TNFA *and* TGFB) and a shift in macrophage phenotype towards Ly6C^high^. Mechanistically, MST1 activation by soluble egg antigen (SEA) promoted PPARγ-mediated CD36 expression, enhancing phagocytosis and consequently upregulation of fibrolytic genes such as *Arg1* and *Mmps*. Conversely, MST1 deletion leads to up-regulation of pro-inflammatory genes instead of fibrolytic genes in macrophages, accompanied by decreased expression of CD36 and impaired phagocytosis. Furthermore, the ablation of MST1 enhances NF-κB activation in *S*. *japonicum*-infected and SEA-stimulated macrophages, resulting in increased production of proinflammatory cytokines. Overall, our data identified MST1 as a novel regulator for egg-induced liver fibrosis via modulation of macrophage function and phenotype by CD36-mediated phagocytosis and suppression of NF-κB pathway.

## Author summary

Macrophages are pivotal in the pathogenesis and resolution of liver fibrosis, particularly in the context of Schistosoma-induced disease. Our study reveals that MST1 (also known as STK4), a serine/threonine kinase, is downregulated in macrophages from mice infected with schistosomes. In a murine model of schistosomal infection, we observed a pronounced exacerbation of liver fibrosis in animals with a targeted Mst1 gene deletion in macrophages.

For a long time, it was believed that liver fibrosis was irreversible, however, recent studies have shown that liver fibrosis and even cirrhosis are reversible. Macrophages play an important role in the development and regression of liver fibrosis, including that caused by Schistosoma infection. Here, we found that MST1 (also STK4), serine/threonine kinase, is downregulated in macrophages from mice infected with schistosome. In a murine model of schistosomal infection, we observed a pronounced exacerbation of liver fibrosis in mice with macrophages specific *Mst1* knockout. We found that MST1 influences the phenotype and function of macrophages through various mechanisms. Firstly, it attenuates the inflammatory response by inhibiting the NF-κB pathway, thereby reducing the production of inflammatory cytokines. Secondly, MST1 enhances the expression of CD36 on macrophage through PPARγ phosphorylation at Ser273. Through CD36-mediated phagocytosis, macrophages undergo a phenotypic transition from a pro-inflammatory and profibrotic phenotype to one that actively facilitates the resolution of fibrosis, thereby protecting against schistosomiasis-induced liver fibrosis. Our study provides valuable evidence for MST1 in orchestrating macrophages functions towards anti-inflammatory and antifibrotic response and may have implications for the development of novel therapeutic strategies.

## Introduction

Schistosomiasis is an endemic and debilitating parasitic disease caused by Schistosoma and currently afflicts more than 200 million people in 78 countries [1]. Schistosome eggs induce macrophages-rich granulomas and Th2-based immune response that precipitates liver fibrosis and eventually cirrhosis in chronically infected hosts. Liver cirrhosis and portal hypertension are the primary causes of chronic morbidity and mortality in schistosomiasis [2]. Excitingly, both animal models and clinical data have shown that liver fibrosis and even cirrhosis are reversible [3]. This has clear clinical significance for therapeutic interventions that leverage this inherent plasticity.

Studies from Schistosome infection-induced liver fibrosis revealed that alternatively activated macrophages (AAMs) compose about 20% of egg-induced liver granulomas, suggesting AAMs play an important role in hepatic fibrosis [4]. Indeed, it has been demonstrated that pro- and antifibrotic roles for AAMs in infected mice. There is growing interest in deciphering the unique set of genes that drive macrophages to exhibit anti-inflammatory and antifibrotic activity, such as *Arg1* [5] and *Retnla* [6]. While some studies have described these molecules as pro-fibrotic, recent evidence suggests they may also exhibit anti-fibrotic properties under certain conditions. For instance, Arg1 has been shown to promote the degradation of extracellular matrix components through the production of polyamines, thus potentially limiting fibrosis [5,7]. In addition, phagocytosis by macrophages has recently emerged as an important mechanism for the polarization of anti-inflammatory macrophages [8–10]. The polarization of macrophages towards an anti-inflammatory phenotype, mediated by phagocytosis through scavenger receptors like CD36 and Stablin-1, has emerged as a critical mechanism that influences fibrosis progression. The impact of phagocytosis on fibrosis is, however, contingent upon the type of dead cells engulfed and removed by the macrophages [11].

Anti-fibrotic AAMs, also known as restorative AAMs, express a unique set of genes that are pivotal for tissue repair and inflammation resolution, including Arg1, IL10, Matrix Metalloproteinases (MMPs), and CD36. On the contrary, pro-fibrotic AAMs display a gene expression profile that amplifies fibrotic responses, characterized by elevated levels of TGFβ, TNFα, and IL1β [8,12].

The scientific community is increasingly interested in understanding the unique gene set that propels macrophages to exhibit anti-inflammatory and anti-fibrotic activities. The anti-fibrotic AAMs and pro-fibrotic AAMs here do not belong to the classic AAMs and possess unique markers. However, the knowledge on signals that instruct AAMs to adopt an anti-inflammatory and an antifibrotic phenotype is very limited.

Mammalian Ste20-like kinase 1, MST1 also known as STK4 (serine/threonine-protein kinase 4) is the key kinase of Hippo pathway. In the canonical Hippo pathways, MST1 functions in the regulation of cell growth and apoptosis by suppressing the transcription factor YAP [13]. Recently, MST1 was found to modulate immune response and inflammation by cross-talking with other signaling pathways in various immune cells [14]. In particular, MST1 was shown to inhibit TLRs-mediated production of proinflammatory cytokines (TNFA, IL6 and IL1B) and profibrotic gene expression in a chronic live fibrosis model via modulating activity of related transcription factors [15]. SEA of *Schistosoma* is known to signal through TLR2/4 on macrophage and modulates granulomatous inflammation and subsequent liver fibrosis [16]. However, the effects of MST1 on the phenotype of macrophages in schistosomiasis-induced liver fibrosis are still unclear.

In the present study, we found that MST1 protects against liver fibrosis caused by *Schistosoma* infection. Macrophages-specific *Mst1* knockout mice exhibit worsened liver pathology with larger granulomas and increased fibrosis. Mechanistically, genetic ablation of MST1 reduced the expression of CD36 and consequently impaired CD36-mediated phagocytosis, which skewed macrophages to a pro-inflammatory phenotype. Moreover, MST1 ablation also enhances NF-κB activation increasing proinflammatory cytokine production. Altogether, our data has identified MST1 as a novel regulator for egg-induced liver fibrosis via modulation of macrophage function and phenotype. This suggests that the enhancement of MST1 activity could potentially offer therapeutic benefits for liver fibrosis.

## Results

### MST1 is downregulated in macrophages from fibrotic livers of *S*. *japonicum*-infected mice and SEA-treated RAW264.7 cells

**Fig 1:**
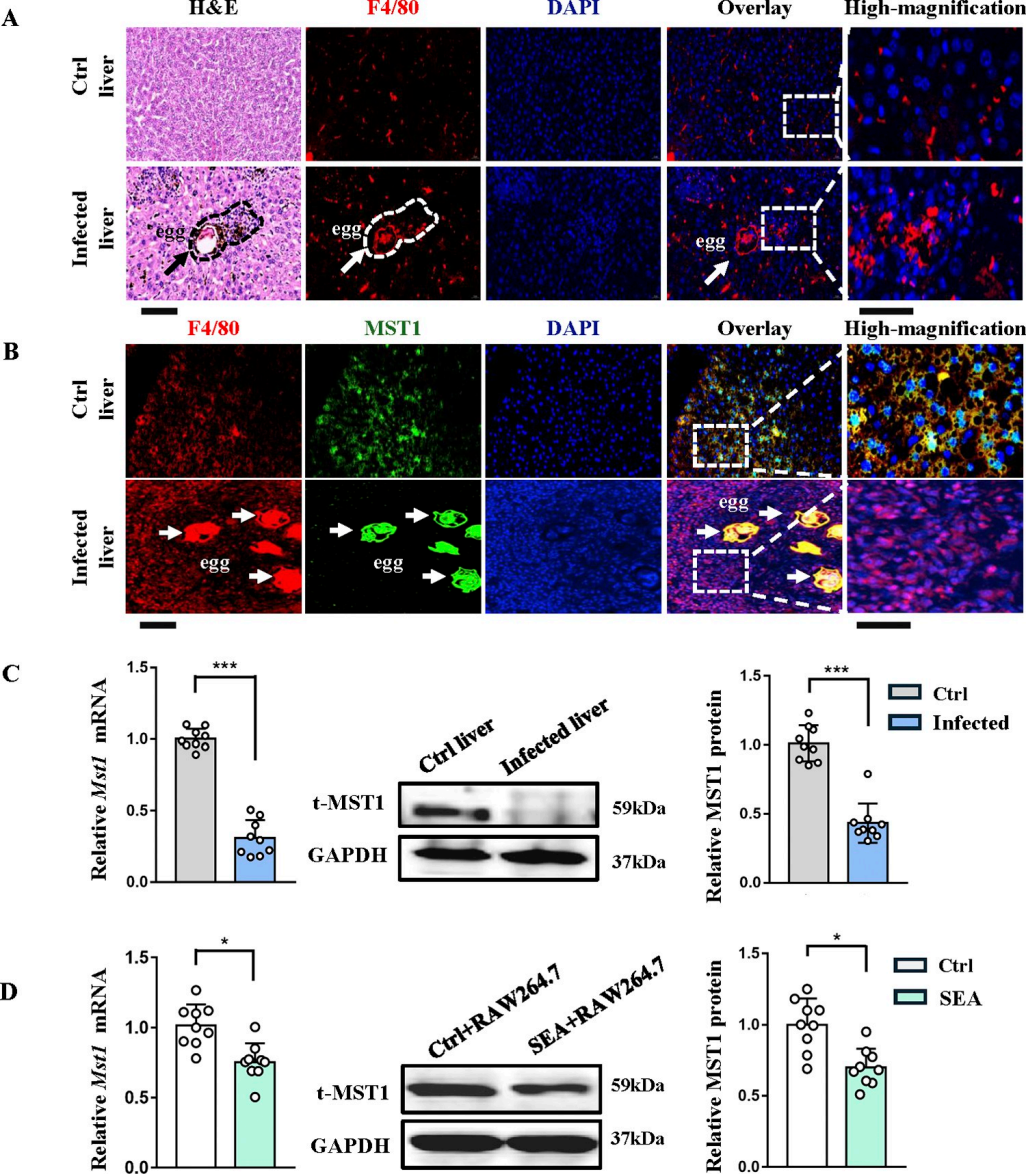
MST1was downregulated in macrophages from fibrotic livers of *S*. *japonicum*-infected mice and SEA-treated RAW264.7 cells. Female mice were infected percutaneously with *S*. *japonicum* cercaria (20±2)and killed for tissue collection at 12w p.i. along with age- matched control mice. (A) Liver sections (4 μm) from infected and control mice stained with HE and immunofluorescent staining of F4/80 and DAPI (Bar of the left four panels = 20 μm, bar of the far right panel = 10 μm). (B) Dual immunofluorescence for F4/80 and MST1 in liver from infected and control mice. Arrows in A and B denote eggs (Bar of the left four panels = 20 μm, bar of the far right panel = 10 μm). (C, D) Relative *Mst*1 mRNA level and protein level in liver tissues from infected and control mice (C) and RAW264.7 cells treated with or without SEA (50μg/ml, 24h) (D) assessed by qPCR and immunoblotting, respectively. Values are mean ± SEM (n = 9, pooled from 3 independent experiments, 3 mice per group for each experiment). Statistical significance was determined by student’s *t* test (C-D). *P < 0.05, ***P < 0.001.

TLRs-signaling-triggered inflammation has been reported to downregulate MST1 in macrophages [17]. We wondered whether MST1 is downregulated post schistosome infection because SEA acts through TLR2/4 signaling and is important in Schistosoma egg-induced liver fibrosis. In agreement with our previous report, 12 weeks after *S*. *japonicum* infection, the C57BL/6 mice developed chronic liver fibrosis [18]. HE staining of liver sections from infected mice showed significant immune cell infiltration in granulomas around schistosome eggs (Fig 1A, the leftmost panel). By immunostaining analysis using anti-F4/80^+^, we confirmed the enhanced infiltration of macrophages within egg-induced fibrotic granulomas (Fig 1A, the right four panels). However, MST1 expression in infiltrating F4/80^+^ cells were significantly lower than that in macrophages from uninfected livers (Fig 1B). Consistently, reduced mRNA and protein levels of MST1 were observed in the fibrotic livers induced by *S*. *japonicum* infection (Fig 1C). In addition, when RAW264.7 cells were treated with SEA (50 μg/ml, 24h), to mimic *S*. *japonicum* infection, we observed a significant reduction of MST1 protein by immunoblotting (Fig 1D). These in vivo and in vitro data show that *S*. *japonicum* infection downregulates the expression of MST1 in macrophages, suggesting MST1 may play a regulatory role in schistosomiasis-induced liver fibrosis.

### *S*. *japonicum* infection activates MST1 kinase activity

**Fig 2:**
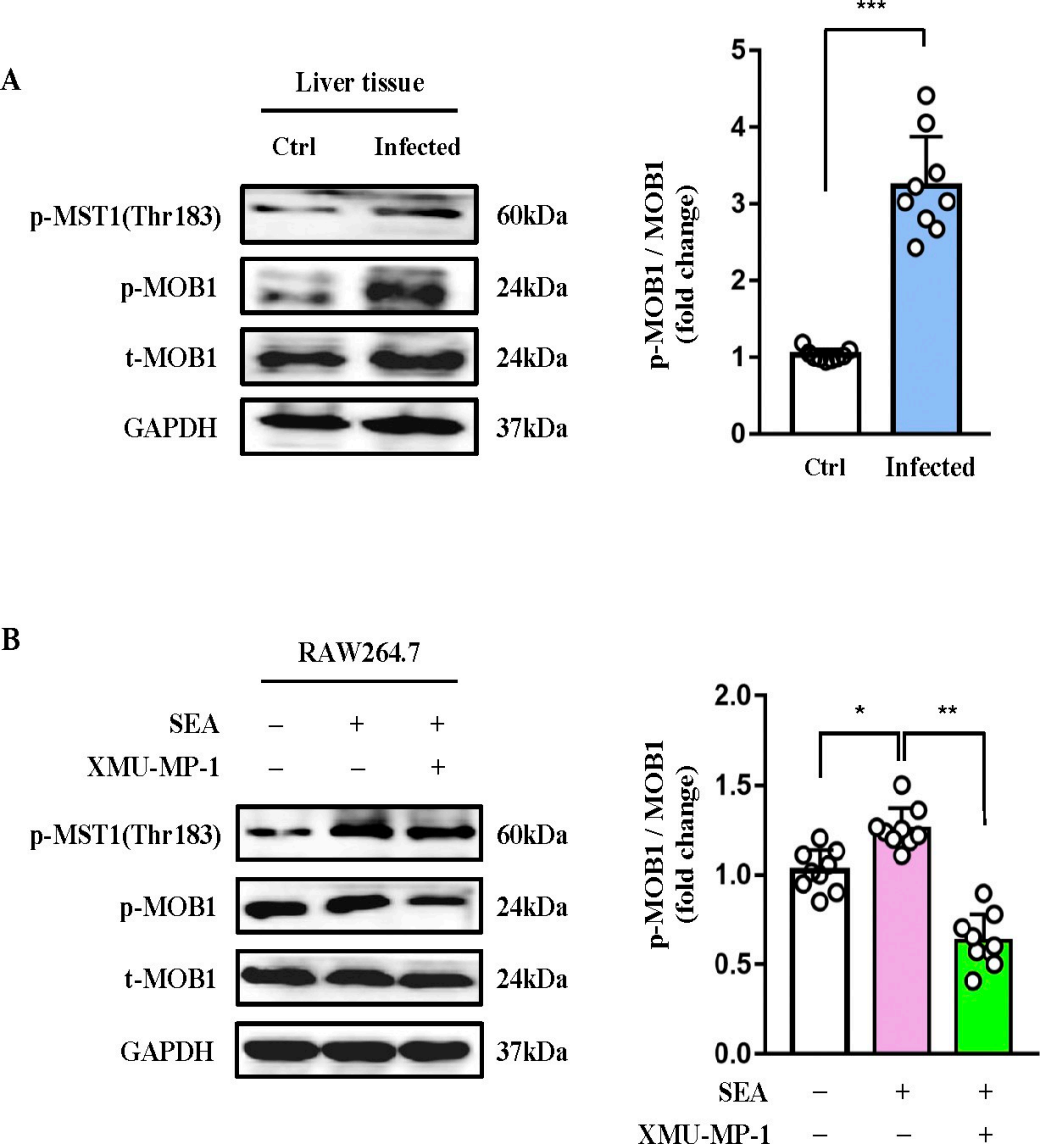
*japonicum* infection induces MST1 activation. ***S*.** (A, B) Immunoblot analysis of phosphorylated (p-) MOB1, total MOB1 and GAPDH (loading control throughout) in liver tissues from *S*. *japonicum*-infected (12w p.i.) and control mice (A) and in RAW264.7 cells untreated or treated with SEA ± XMU-MP-1 (B). Quantitative analysis of p-MOB1 levels relative to corresponding total proteins. Values are mean ± SEM (n = 9, pooled from 3 independent experiments, 3 mice per group for each experiment). Statistical significance was determined by Student’s *t* test (A) and one-way ANOVA analysis (B). *P < 0.05, **P < 0.01, ***P < 0.001.

MST1 has been reported to participate in TLR-mediated activation of macrophages, multiple evidence suggests that SEA activates macrophages via TLRs [17,19,20]. To determine whether the kinase activity of MST1 is involved in *S*. *japonicum* infection, we examined phosphorylated MST1 and MOB1, a physiological substrate of MST1. Unexpectedly, the phosphorylation levels of MST1 and MOB1 in liver from *S*. *japonicum-*infected mice were higher than those from uninfected mice (Fig 2A). In agreement with this, SEA stimulation substantially enhanced the phosphorylation of MOB1 in RAW264.7 cells. Treatment of the cells with MST1 kinase inhibitor XMU-XP-1 reduced the phosphorylation of MOB1 (Fig 2B). Thus, increased phosphorylation of MST1 and MOB1 suggests MST1 kinase activation by *S*. *japonicum* infection.

### *Mst1*^*△M/△M*^ mice develop exacerbated liver fibrosis pathology after *S*. *japonicum* infection

**Fig 3:**
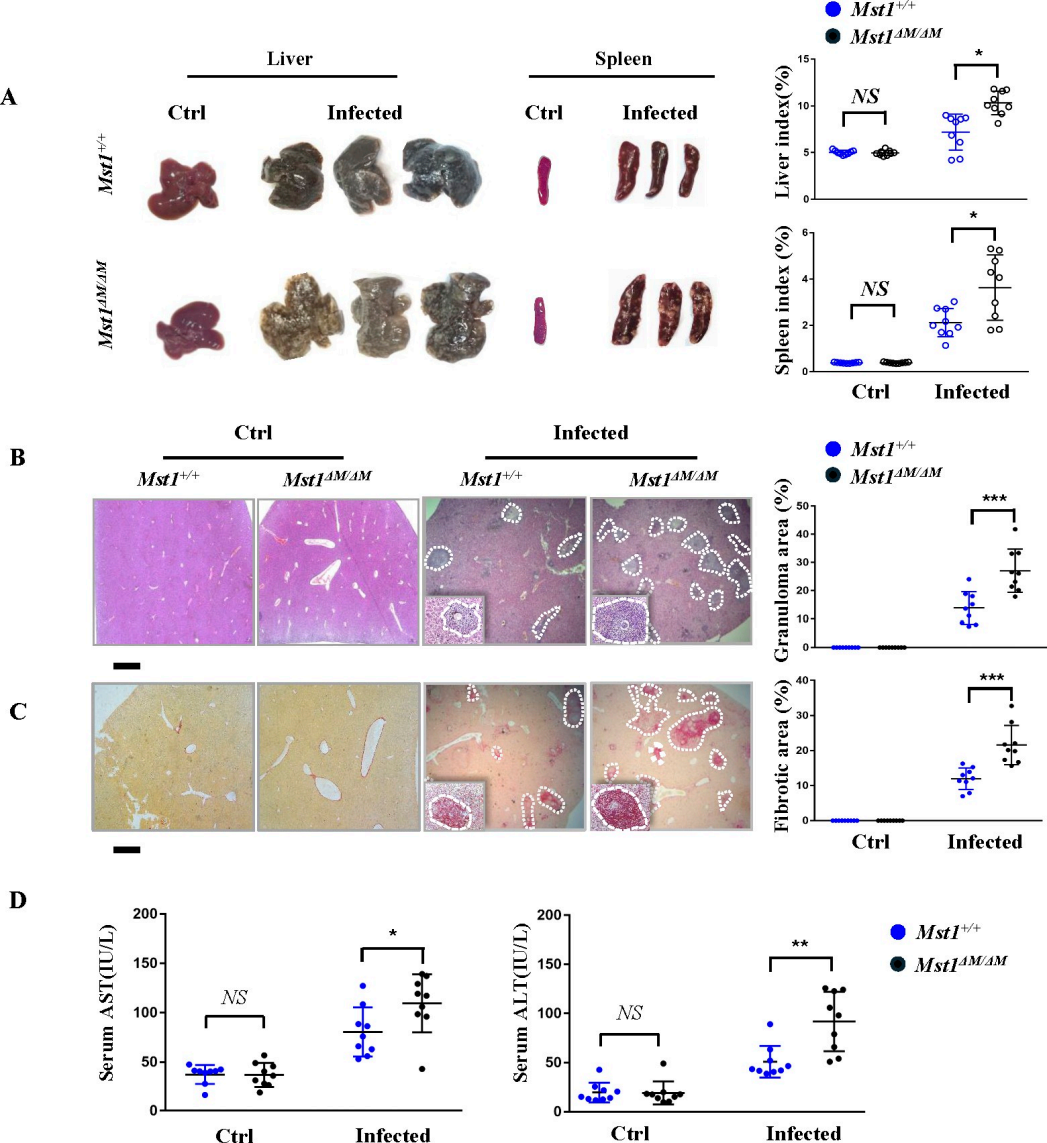
*Mst1*^*△M/△M*^ mice develop exacerbated liver fibrosis pathology after *S*. *japonicum* infection. Livers, spleens and serum were collected from control and *S*. *japonicum-*infected (week 12 p.i.)*Mst1*^*+/+*^ and *Mst1*^*ΔM/ΔM*^ mice. Liver sections (4 μm) were stained with Hematoxylin and Eosin (HE) and Sirius Red. Representative images of livers and spleens from Mst*1*^*+/+*^ and *Mst1*^*ΔM/ΔM*^ mice with liver/body and spleen/body ratio are shown in (A). HE staining and Sirius Red staining of liver sections of the indicated groups are displayed in (B) and (C) respectively. Percentages of granulomatous and fibrosis areas were quantified and are shown on the right side in (B) and (C) respectively (Bar = 200 μm). Magnification was 40×. Representative images of single eggs are shown in the insets. (D) Levels of ALT and AST in the serum from control and infected *Mst1*^*+/+*^ and *Mst1*^*ΔM/ΔM*^ mice were detected. Values are mean ± SEM (n = 9, pooled from 3 independent experiments, 3 mice per group for each experiment). Each circle represents a value from individual mouse. All *p* values were determined by Student’s *t*-test *vs* Mst1^+/+^ mice. *P < 0.05, **P < 0.01, ***P < 0.001.

To investigate whether MST1 plays a direct role in egg-induced liver fibrosis, we produced myeloid (including macrophages) specific *Mst1* knockout mice (termed *Mst1*^*△M/△M*^ mice) by crossing *Mst1*^*flox/flox*^ mice with *LysMCre* mice. The knockdown efficiency of *Mst1* was confirmed, while the knockdown of MST1 showed no significant effect on the major immune cell populations in the liver. *Mst1*^*flox/flox*^ (also called *Mst1*^*+/+*^ or WT mice here) and *Mst1*^*△M/△M*^ mice were infected percutaneously with 20 ± 5 cercariae to constructed *S*. *japonicum*-induced liver fibrosis model as previously reported [18,21]. Mice were sacrificed at 12 weeks post infection (p.i.). Compared with the WT mice, *Mst1*^*△M/△M*^ mice displayed more severe liver pathology. A striking feature of the *Mst1*^*△M/△M*^ mice liver was the larger yellowish white egg granulomatous nodules on the surface from gross photographs (Fig 3A, left panel), which are generally present at acute time-point (7 to 9 weeks p.i.) in WT mice. A significantly higher liver and spleen index of *Mst1*^*△M/△M*^ mice are consistent with the severity of liver pathology (Fig 3A, right panel). Egg-induced granulomatous inflammation and fibrosis were enhanced in *Mst1*^*△M/△M*^ mice shown by HE and Sirius Red staining, respectively (Fig 3B and 3C). We also detected more liver injury in *Mst1*^*△M/△M*^ mice, as determined by serum by aminotransferase ALT and AST levels (Fig 3D). In addition, no difference in the number of eggs in the livers was detected between WT and *Mst1*^*△M/△M*^ mice (S1 Fig). Collectively, *S*. *japonicum-*infected mice model demonstrates the protective role of MST1 in macrophages during egg-induced liver fibrosis.

### *Mst1* knockout increases proinflammatory phenotype of macrophages from *S*. *japonicum*-infected mice and upregulates the expression of profibrotic genes

**Fig 4:**
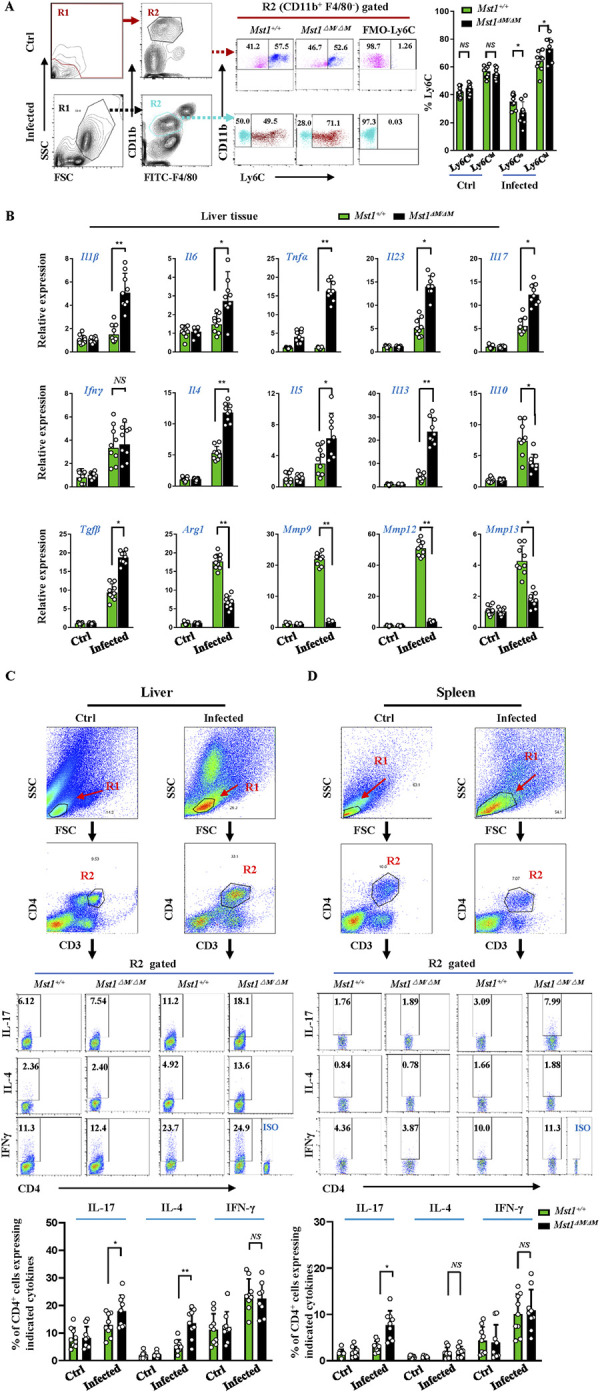
Mst1 knockout increases proinflammatory phenotype of macrophages and Th17 response from *S*. *japonicum-*infected mice. (A)Liver mononuclear cells from control mice and liver granuloma cells from *S*. *japonicum-*infected *Mst1*^*+/+*^ and *Mst1*^*ΔM/ΔM*^ mice (week 12 p.i.) were isolated for flow cytometry analysis. Gating strategy (left panels): Dead cells were excluded by DAPI stain (displayed in S2 Fig). R1 (myeloid cell population) was gated based on forward scatter (FSC) and side scatter (SSC). Within R1, the CD11b-positive and F4/80-negative cells are gated as R2. The proportions of Ly6C^high^ and Ly6C^low^ were analyzed within R2. The representative percentages of Ly6C^high^ and Ly6C^low^ are displayed in the middle panels. The numbers represent the frequency of the indicated population within the CD11b^+^ cells. (B) The abundance of mRNA of indicated genes in livers from control and infected *Mst1*^*+/+*^ and *Mst1*^*△M/△M*^ mice was analyzed by qPCR.(C, D) Flow cytometry analysis of IL17^+^, IL4^+^ and IFNγ^+^ in CD4 T cells from the livers (C) and spleens (D) of infected and control mice stimulated with PMA and ionomycin in vitro. After excluding dead cells (S2 Fig), R1 (lymphocyte gate) was gated based on FSC and SSC. R2 represented CD3^+^CD4^+^ T cells gated from R1. IL-17-, IL-4-, and IFNγ-producing cells were analyzed from R2 (upper panels). The representative percentages of IL17^+^, IL4^+^, IFNγ^+^ cells within this gate were shown in middle panels of C and D, with the isotype controls for the staining antibodies shown in the lower right corner insets. Statistical quantification of the expression of IL17^+^, IL4^+^, IFNγ^+^ cells in the liver and spleen of control and infected *Mst1*^*+/+*^ and *Mst1*^*△M/△M*^ mice is presented in the bottom panels of C and D, respectively. Data are presented as mean ±SEM (n = 9, pooled from 3 independent experiments, with 3 mice per group for each experiment). Each circle represents an individual mouse value, *P < 0.05, **P < 0.01, All *P* values were determined by Student *t*-test vs Mst1^+/+^ mice.

Next, we proceed to the effect of *Mst1* knockout on phenotypic changes in liver macrophages from *S*. *japonicum*-infected mice (week12 p.i.). The CD11b-positive subset (including Ly6C^high^ and Ly6C^low^) represents a recruited monocyte-derived macrophage population, which has been shown to play an intricate role in egg-induced liver fibrosis [22]. Flow analysis of lymphocytes from fibrotic livers revealed a significantly higher proportion of Ly6C^high^ in *Mst1*^*△M/△M*^ mice compared to their WT counterparts (Fig 4A). Consistent with the macrophage responses, qPCR analysis of the liver tissue from *S*. *japonicum-*infected *Mst1*^*△M/△M*^ mice revealed significant upregulation of pro-inflammatory (Il*1b*, *Il6*, *Tnfa*, *Il23 and Il17*), and profibrotic genes (*Tgfb Il4*, *Il5 and Il13)*, while the expression of antifibrotic genes (*Il10*, *Arg1 and Mmps*) was significantly diminished (Fig 4B). In addition, MST1 deficiency led to a marked increase in the expression of IL17 and IL4, but similar IFNγ in liver CD4 T cells (Fig 4C). In the spleen, CD4 T from *Mst1*^*△M/△M*^ mice had more IL17^+^ cells, while there is no significant difference in IL4^+^ and IFNγ^+^ cells between the two groups (Fig 4D). These findings collectively suggest that MST1 deficiency boosts Th2 and Th17 responses in the livers of schistosome-infected mice. The increased proportion of Ly6C^high^ monocyte-derived macrophage, along with the significant upregulation of pro-inflammatory and profibrotic genes, and the downregulation of antifibrotic genes, point towards a pro-inflammatory liver environment that may contribute to the exacerbation of fibrosis in these mice. The lack of difference in IL-4^+^ cells in the spleen indicates that the Th2 response is more localized to the liver, further emphasizing the liver-specific nature of the immune response in the context of MST1 deficiency.

### Mst1 knockout downregulates the expression of CD36 in PEMs and liver macrophages

**Fig 5:**
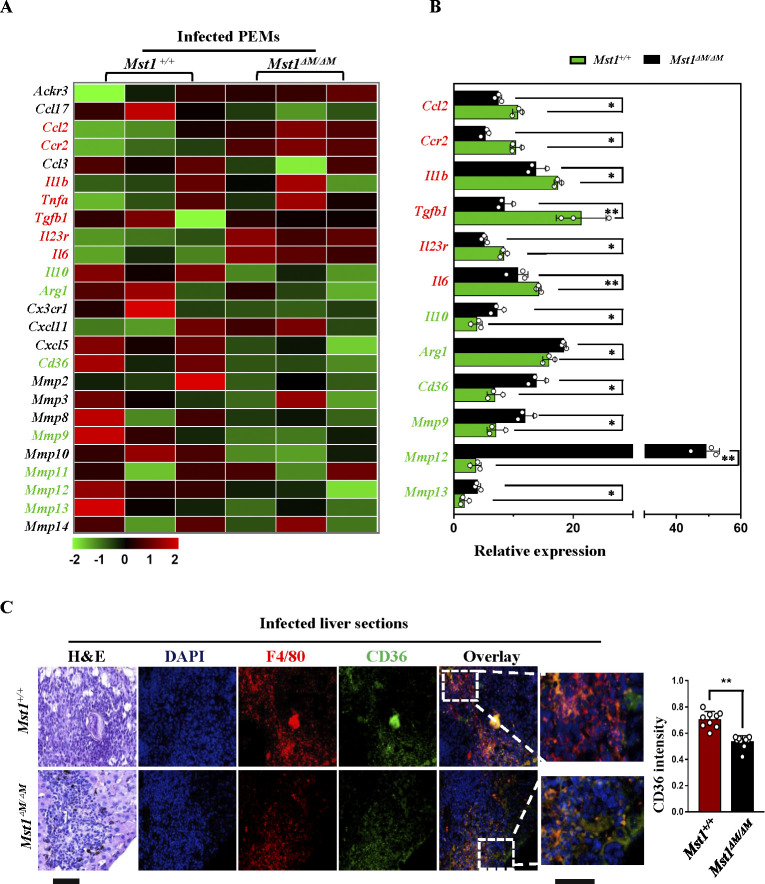
Mst1 knockout skewed macrophage polarization toward a proinflammatory and profibrotic phenotype with concomitant down-regulation of CD36. PEMs and liver tissues were collected from *Mst1*^*+/+*^ and *Mst1*^*ΔM/ΔM*^ mice infected with 20 ± 5 cercariae and sacrificed at week 12. (A) RNA-seq analysis was performed on differentially expressed genes (DEG) in PEMs from infected *Mst1*^*+/+*^and *Mst1*^*ΔM/ΔM*^ mice control *Mst1*^*+/+*^and *Mst1*^*ΔM/ΔM*^ mice (n = 3). DEGs were identified in all pairwise comparisons and showed a 2-fold change in expression with an adjusted *p-*value of 0.05. (B) Some DEGs were verified by qPCR in PEMs from infected mice. (C) Liver sections from infected mice were subjected to HE staining (leftmost panel) and dual immunofluorescence for CD36 and F4/80 (2–5 panels on the right side) in. High-magnification images of co-staining for F4/80 and CD36 cells were shown in the right most panel. The fluorescence intensity of CD36 overlaid with F4/80^+^ cells was analyzed using ImageJ. Data are shown as mean ± SEM of three different liver sections in each mouse (n = 9, data pooled from 3 independent experiments, 3 mice per group for each experiment. Bar of the left five panels = 100 μm, bar of the far right panel = 50 μm).All *p* values were determined by Student’s *t*-test. *P < 0.05, **P < 0.01.

To explore how MST1 protects against schistosome liver fibrosis, RNA sequencing (RNA-seq) was performed in peritoneal macrophages (PEMs) from *Mst1*^*+/+*^ and *Mst1*^*△M/△M*^ mice infected with schistosome (12w p.i.). A total of 236 genes were upregulated in *Mst1*^*△M/△M*^ macrophages. As expectedly, several canonical proinflammatory genes implicated in fibrosis, *Ccl2*, *Ccr2*, *Il1b*, *Tgfb*, *Il6 and Il23r* were significantly upregulated in *Mst1* knockout macrophages, while some key fibrolytic genes, such as *Il10*, *Arg1*, *Cd36 and Mmps* were significantly reduced (Figs 5A and S3). These genes showed no differences between the control *Mst1*^*+/+*^ and *Mst1*^*△M△M*^ mice, except for MMP13 (upregulated control but downregulated *in infected Mst1*^*△M△M*^ mice S3 Fig). Some differentially expressed genes (DEGs) were further validated by quantitative real-time PCR (qPCR) (Fig 5B). We also noted that scavenger receptor CD36 was also significantly decreased in *Mst1*-deficient macrophages (Fig 5A and 5B). To determine whether *Mst1* knockout has the same effect on CD36 expression of macrophages in vivo, confocal microscopy was performed on infected liver sections. We observed a significant proportion of the F4/80^+^population are CD36 positive across granulomas on the WT liver sections, however, CD36^+^ frequency in F4/80 positive area was significantly lower in *Mst1*^*△M/△M*^ livers (Fig 5C). Taken together, these findings suggest that endogenous MST1 inhibits the proinflammatory phenotype of macrophages, and *Mst1* deficiency reduces CD36 expression in macrophages from *S*. *japonicum-*infected mice.

### Downregulation of CD36 impaired phagocytic capacity and is associated with a proinflammatory phenotype of *Mst1* deficiency macrophages

**Fig 6:**
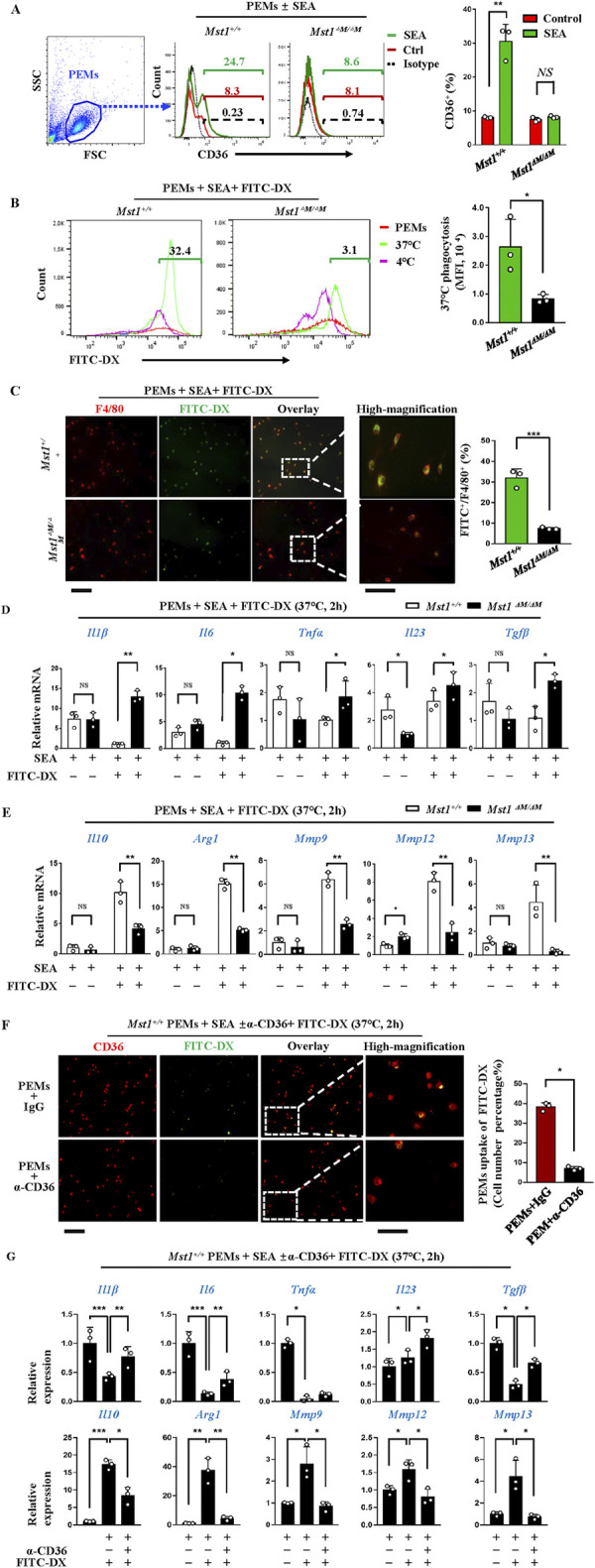
Downregulation of CD36 impaired phagocytic capacity and is associated with a proinflammatory phenotype of *Mst1* deficiency macrophages. WT or *Mst1* deficiency peritoneal exudate macrophages (PEMs) were stimulated with or without SEA (50μg/ml) for 24h, followed by incubation with FITC-Dextran for 2 h. (A) Flow cytometry analysis of the proportion of CD36^+^ cells. Isotype-matched control IgG staining (----); anti-mouse CD36 staining (——, no SEA;——, with SEA) The representative percentage of CD36 positive cells are shown in the left panels. Gating strategies are shown in the left panels. (B) Flow cytometry analysis of the proportion of PEMs engulfing FITC-dextran, with analysis of mean fluorescence intensity (MFI) in engulfed PEMs. (C) Immunofluorescence assay of PEMs engulfing FITC-dextran, with representative images from three separate cell isolates (magnification×200) and percentages of FITC^+^ in F4/80^+^ PEMs shown. Ten viewing fields were analyzed in each experiment(Bar of the left three panels = 40 μm, bar of the far right panel = 20 μm). (D, E) Changes in gene expression in PEMs after the uptake of FITC-dextran (37°C, 2h). (F, G) SEA cultured WT PEMs were pretreated with isotype control (clone EPR25A, ab172730) and or CD36 function-blocking antibody (clone EPR22509-40, ab252922), followed by incubation with FITC-Dextran (2 h, 37°C), and subjected to immunofluorescent staining analysis. Representative images were shown (magnification×200) on left panels. Percentages of coimmunostaining of CD36^+^ and FITC^+^ PEMs were shown on the right side (Bar of the left three panels = 40 μm, bar of the far right panel = 20 μm) (F). mRNA expressions of indicated genes were detected by qPCR (G). Data are shown as mean ± SEM (n = 3, three samples consisting of pooled PEMs of two or three mice from representative of three independent experiments). Statistical significance was determined by student *t* test (A-F) and one-way ANOVA analysis (G). *P < 0.05, **P < 0.005, ***P < 0.001.

As phagocytosis induces phenotypic switch from fibrogenic macrophages to restorative and fibrolytic macrophages [23,24]. To investigate the effect of CD36-mediated phagocytosis on macrophages phenotype, we examined the expression pattern of known anti-, pro-inflammatory and anti, profibrotic cytokines in PEMs that phagocytosed FITC-dextran. WT or *Mst1* deficiency PEMs were stimulated with SEA (50 μg/ml) for 24 hours, followed by FITC-Dextran incubation for 2 h. SEA stimulation of WT PEMs led to a significant increase in CD36 expression, whereas no significant expression of CD36 was detected in *Mst1* deficiency PEMs (Fig 6A). By flow cytometry analysis, uptake of FITC-Dextran was minimal at 4°C, but greatly enhanced at 37°C in WT PEMs, as shown by the increased proportion of FITC^+^ populations. In contrast, FITC^+^ populations were significantly lower in *Mst1* deficiency PEMs, suggesting a weaker phagocytic capacity (Fig 6B). This was further confirmed by immunofluorescence imaging analysis, a significant reduction in the co-localization of FIITC and F4/80 in *Mst1* deficiency PEMs (Fig 6C). This data indicated that *Mst1* deficiency substantially impaired phagocytic capacity along with reduced CD36 expression. Furthermore, the abundance of the proinflammatory (e.g. *Il1b*, *Il6*, *Il23*, *Tnfa*) and profibrotic (*Tgfb*) genes were higher in *Mst1* deficiency PEMs after phagocytosis of dextran (Fi 6D). On the contrary, we observed that WT PEMs phagocytosis of FITC-Dextran resulted in a significant up-regulation of genes associated with anti-inflammatory or antifibrotic effects (e.g. *Il10*, *Mmp9*, *Mmp12*, *Mmp13*, *Arg1*). However, all five genes were not significant up-regulation in *Mst1* deficiency PEMs (Fig 6E). Importantly, no significant difference in the expression of these genes between WT and *Mst1* deficiency PEMs was observed before phagocytosis. Consistent with the results in *Mst1* deficiency PEMs, reduced ability to FITC-Dextran uptake and similar changes of these genes expression were also observed in WT PEMs pretreated with CD36 blockade as shown by immunostaining and qPCR (Fig 6F and 6G). These results support that CD36-mediated phagocytosis resulted in substantial modulation of macrophage antifibrotic phenotype.

### MST1 regulates the expression of CD36 through PPARγ phosphorylation

**Fig 7:**
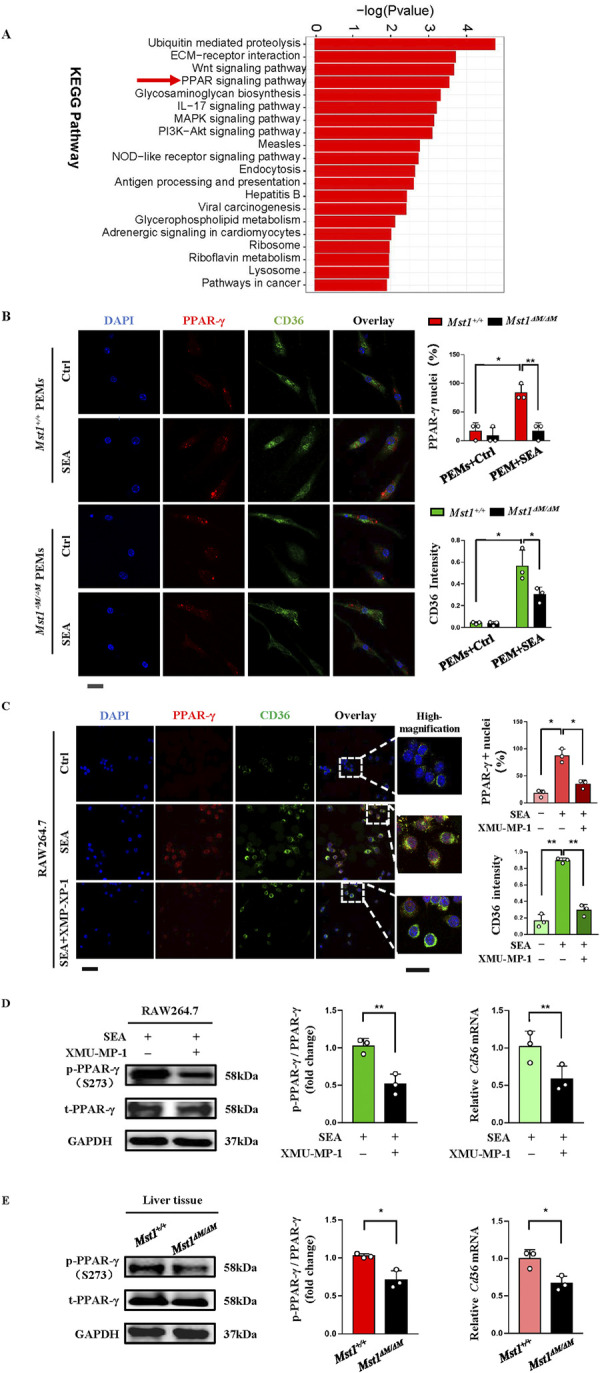
MST1 upregulates CD36 expression via PPARγ. (A) Pathway analysis of the RNA-Seq data comparing PEMs from Mst1^+/+^ and Mst1 ^ΔM/ΔM^ mice infected by *S*. *japonicum* for 12 weeks. (B) Immunofluorescence assay of SEA (24 h, 50μg/ml) or control cultured WT and MST1- deficiency PEMs for CD36 and PPARγ. Images were collected with a confocal microscope and CD36 intensity was quantified using ImageJ. Data are shown as mean ± SEM (n = 3, three samples consisting of pooled PEMs of two or three mice from representative of three independent experiments. Bar = 10 μm). (C) Immunofluorescence assay for CD36 (green), PPARγ (red) in RAW264.7 cells stimulated with or without SEA (24 h, 50μg/ml), followed by XMU-MP-1 incubation (3μM, 6h). Images were taken with a confocal microscope. Percent nuclear PPARγ fluorescence and CD36 intensity in macrophages with PPARγ localization were analyzed using ImageJ. Data from at least three independent experiments, with 10 viewing fields were analyzed in each (Bar of the left four panels = 10 μm, bar of the far right panel = 5 μm). (D, E) Immunoblot analysis of phosphorylated (p-)PPARγ and total PPARγ (left and middle) and relative *Cd36* mRNA (right) in RAW264.7 cells treated with SEA ± XMU-MP-1(D) and in liver tissues from *Mst1*^*+/+*^ and *Mst1*^*ΔM/ΔM*^ mice infected and control mice (E). Values are means ± SEM (n = 3, from representative of three independent experiments). Statistical significance was determined by Mann-Whitney U-test (B), one way ANOVA analysis (C) and Student’s *t* test (D-E). *P < 0. 05, **P < 0. 01.

Scavenger receptor CD36 has been identified as a target gene of PPARγ in macrophages, and KEGG analysis of DEGs between *S*. *japonicum*-infected WT and *Mst1*^*△M/△M*^ PEMs suggested PPAR signal pathway enriched (Fig 7A). The finding that *Mst1* deficiency impaired CD36 expression in macrophages indicated a possible link between the MST1 and the transcriptional activation PPARγ. To investigate this possibility, WT and *Mst1*-deficient PEMs were purified and stimulated with or without SEA (50 μg/ml) for 24 hours. Confocal imaging revealed that a low level of background cytoplasmic staining but no a nuclear staining of PPARγ was seen in control WT PEMs. A punctate nuclear staining of PPARγ was observed in SEA stimulated WT PEMs as well as significantly enhanced CD36 staining (Fig 7B, the upper two panels). However, in *Mst1*-deficient PEMs, SEA treatment had little effect on CD36 expression and nuclear staining of PPARγ (Fig 7B, the bottom two panels). These findings indicated that MST1 regulates CD36 by a PPARγ dependent mechanism.

The S273 phosphorylation of PPARγ by p21-activated kinase 4 (PAK4) increases its transcription activity [24]. We next investigated whether MST1 kinase activity was necessary for PPARγ dependent CD36 expression. Immunofluorescence observations of RAW264.7 cells stimulated with SEA (50 μg/ml, 24h) showed a striking increase of PPARγ staining in nuclear and CD36 signal on the member (Fig 7C, middle panel). This activity was strongly inhibited by treatment of the cells with XMU-XP-1 (Fig 7C, bottom panel). Consistently, the reduced S273 phosphorylation of PPARγ and CD36 mRNA (Fig 7D) were also observed by Western blotting and qPCR, respectively. Further, the levels of p-PPARγ at S273 are significantly reduced in the liver tissue from *S*. *japonicum*-infected *Mst1*^*△M/△M*^ mice, confirming that phosphorylation at Ser 273 of PPARγ is occurring specifically through MST1 in *vivo* (Fig 7E). This data indicated that MST1 kinase activity is critical to regulate CD36 expression via the phosphorylation of PPARγ.

### *Mst1* knockout or suppressing its kinase activity increases the production of inflammatory mediators by activating the NF-κB pathway

**Fig 8:**
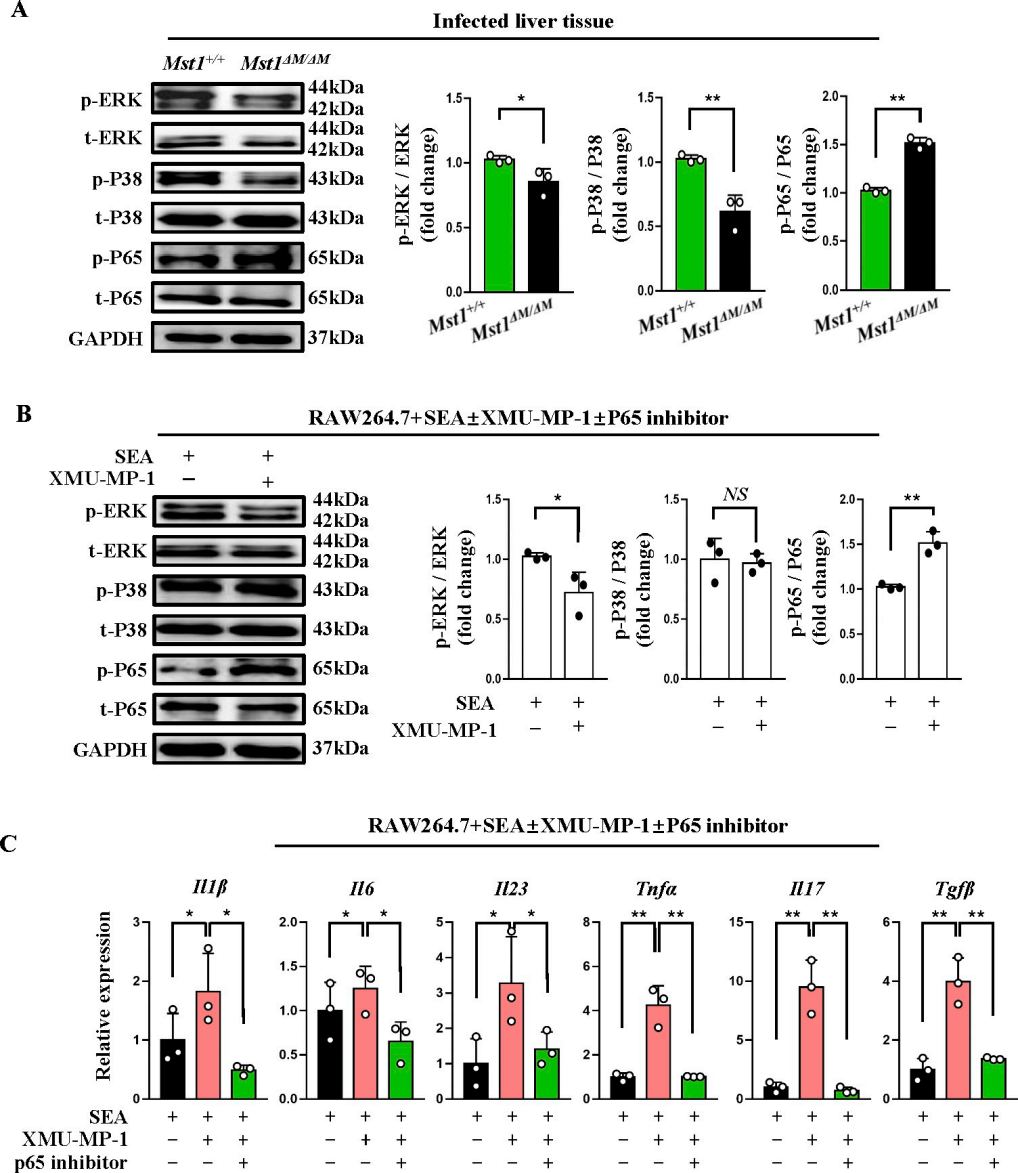
Mst1 knockout or suppressing its kinase activity increases the production of inflammatory mediators by activating the NF-κB pathway. (A,B) Immunoblot analysis of phosphorylation of ERK2, P38, P65 and corresponding total protein in liver tissues from infected *Mst1*^*+/+*^ and *Mst1*^*ΔM/ΔM*^ mice (A), and in SEA- stimulated RAW264.7 cells ± MST1 inhibitor (XMU-MP-1, 3 μM, 6h) (B). Quantification analysis of p-ERK2, p-P38, p-P65 relative to corresponding total proteins. (C) The mRNA of indicated genes in RAW264.7 cells +SEA± XMU-MP-1± P65 inhibitor (BAY 11–7082,1μM, 1h) were analyzed by qPCR. Values are mean ± SEM (n = 3 from representative of from three independent experiments) Statistical significance was determined by student’s *t* test (A-B) and one-way ANOVA analysis (C). *P < 0.05, **P < 0.01.

Given that *Mst1*-deficient macrophages infected with *S*. *japoniucum* skewed to a pro-inflammatory phenotype and SEA signals through TLR2/4, we therefore screened several potential signaling pathways that may be involved in TLR2/4-induced production of pro-inflammatory cytokines in macrophages. We observed that levels of p-P65 were markedly enhanced, while p-ERK2 and p-P38 were reduced in infected liver tissues from *Mst1*^*△M/△M*^ mice versus WT mice (Fig 8A). Consistently, MST1 inhibitor treatment of RAW264.7 cells (SEA stimulated) with XMU-XP-1 significantly increased p-P65 but decreased p-ERK2 (Fig 8B).

We next sought to determine the effect of NF-κB’s phosphorylation level on the expression of inflammatory cytokines of downstream of NF-κB. RAW264.7 cells pretreated with NF-κB inhibitor (1 μM, 1h), in the presence of SEA±XMU-XP-1. As shown in Fig 8C, proinflammatory cytokine, such as *Il1b*, *Il6*, *Il23*, *Tnfa*, *Il17*, *and Tgfb* were repressed by P65-inhibitor. This data indicates that Mst1 knockout or MST1 inhibition promotes the phosphorylation of NF-κB and is at least partially responsible for increasing the production of inflammatory cytokines.

## Discussion

In this study, we reveal a previously undiscovered function of MST1: that of the protective role of MST1 in schistosomiasis-induced liver fibrosis. MST1 phosphorylates PPARγ at S273 residue promoting CD36 expression and subsequently CD36-mediated phagocytosis induced the polarization of egg-AAMs towards an antifibrotic phenotype. Concomitantly, MST1 also inhibits the pro-inflammatory phenotype of macrophages by inhibiting the NF-κB pathway.

MST1 kinase is the core constituent that regulates cell proliferation and survival by inhibiting the transcription factor YAP in the ancient canonical Hippo pathway [25]. YAP has been reported to promote fibrosis by engaging with the TGFβ/Smad pathway, influencing profibrotic gene expression, and potentially forming a positive feedback loop with TNFA in renal fibrosis [26]. Recently, increasing evidence suggested that MST1-dependent noncanonical Hippo pathway regulates the functions of immune cells and plays a critical role in various immune-mediated diseases including autoimmune diseases and fibrosis [14,15,27]. Particularly, MST1 acts as a key negative regulator of TLRs signaling in macrophages, suppressing the release of mediators. Despite macrophages in egg-induced granulomas have long been considered to be AAMs, the fact is that the AAMs in egg-induced granulomas include different two subsets, Ly6C^high^ and Ly6C^low^. Previous studies have shown that both originate from infiltrating Ly6C^high^ monocytes [28]. Ly6C^high^ macrophages exert proinflammatory and profibrotic functions mediated through various inflammatory factors, including TNFA, IL1B and TGFB [29]. In contrast, Ly6C^low^ macrophages play a protective role in anti-inflammatory and antifibrotic processes. The balance between these subsets is crucial for determining the outcome of liver fibrosis, as Ly6C^high^ macrophages can transition into Ly6C^low^ macrophages under certain conditions [30]. Our study revealed that mice lacking MST1 specifically in macrophages exhibited more severe liver fibrosis when infected with *S*. *japonicum*. The number of Ly6C^high^ population is significantly higher in infected livers from *Mst1*^*△M/△M*^ mice compared to WT mice, along with higher levels of inflammatory mediators such as IL6, TNFA, IL1B, IL17 and IL23 in the liver tissue. Previous research by Geng et al. demonstrated that macrophages lacking MST1/2 in display a proinflammatory phenotype characterized by increased levels of IL6, TNFA and IL1B, as well as reduced phagocytic capacities [31]. Li et al. also reported that MST1 inhibits the release of TLR-mediated inflammatory mediators (IL6 and TNFA) in macrophage by interacting with IRAK1, thereby playing a protective role in inflammation-induced hepatocellular carcinoma [15] Simultaneously, according to Tuerxun K and colleagues’ research, the differential expression of MST1, as identified in the study of Echinococcus granulosus infections, suggests its potential significance in the modulation of the host’s immune response and fibrosis, a role that may be analogous to its functions in other helminth infections [32]. This insight lays the groundwork for future research to uncover the precise mechanisms of MST1 in host-parasite interactions, immune responses, and fibrotic development. Future studies might focus on how MST1 influences cytokine production and other immune mediators, as well as its effects on tissue structure and function during infection.. Furthermore, the deletion of dendritic cell MST1 has been shown to increase IL6 expression, leading to the promotion of Th17 differentiation and worsening experimental autoimmune encephalomyelitis [33].

Th17 cells drive liver fibrosis in schistosomiasis by secreting IL-17, which activates TGF-β signaling and collagen deposition, along with TNF-α and IL-1β that boost hepatic stellate cell activation [18]. Th2 cells exacerbate fibrosis through IL-4 and IL-13, promoting fibroblast to myofibroblast transformation and protecting them from apoptosis, thus advancing fibrosis [34]. Our study, aligning with previous research, shows that MST1 knockout intensifies liver fibrosis by enhancing TH17 and Th2 responses.

Interestingly, our findings indicate that in the livers of schistosome-infected mice, there is a downregulation of MST1 protein levels compared to non-infected controls. However, concurrently, we observe an increase in the phosphorylation levels of MST1. This seemingly contradictory observation can be rationalized by considering the known role of MST1 as a suppressor of inflammation. It has been documented in various inflammatory conditions that MST1 protein levels are downregulated, yet its phosphorylation levels are elevated [15]. The expression level of a protein and its phosphorylation level are two distinct regulatory mechanisms. The increased phosphorylation of MST1 in infected animals may represent a compensatory mechanism to enhance its activity, despite the overall reduction in protein levels. This could be an adaptive response by the host to modulate inflammatory responses during infection. Unfortunately, we fail to clarify the mechanism by which schistosome infection or SEA stimulation downregulates the expression of MST1.

Importantly, our findings suggest that inhibiting MST1 kinase activity can reduce Ser 273 phosphorylation and decreased CD36 expression, indicating that MST1 may enhance CD36 expression through PPARγ phosphorylation. Consistently, previous studies have demonstrated that phosphorylation of PPARγ at S273 by Cdk5 and MEK can impact gene expression in adipose tissues, affecting insulin sensitivity [35–37]. Interestingly, PAK4 phosphorylation of S273 has been reported to directly enhances PPARγ’s transcriptional activity in muscle tissue [24]. Although we were unable to elucidate how MST1-mediated S273 phosphorylation regulates the expression of CD36, our observations suggest a correlation between CD36 expression and reduced PPARγ nuclear localization.

By using anti-CD36, we have confirmed that CD36-mediated phagocytic activity leads to the increased expression of fibrolytic genes such as *Il10*, *Arg1 and Mmps*. Previous studies have shown that the scavenger receptor, stabilin-1 protects against liver fibrosis by enabling macrophages to eliminate fibrogenic products of lipid peroxidation [9]. Critically, Iredale et.al. reported that inducing phagocytic behavior in vivo through liposomes administration increases the number of Ly6C^low^ macrophages and accelerates fibrosis resolution. They also observed that phagocytosis of macrophages induces MMPs expression and upregulates a number of PPARγ target genes, including CD36, in Ly6C^low^ subset [23]. Our findings are consistent with these results, as deletion of MST1 in macrophages impairs the upregulation of MMPs and Arg1 following phagocytosis. This is associated with the reduced expression of CD36 and decreased phagocytic ability. Paradoxically, Pennathur S et al. reported that the macrophage CD36 promotes fibrogenic pathways on removal of apoptotic cells during chronic kidney injury. Thus, the function of CD36 can be contextual dependent. Whether phagocytosis promotes or slows the progression of fibrosis depends on the type of dead cells being engulfed and cleared [38].

Additionally, our research revealed that MST1 plays a role negatively regulating the NF-κB pathway during *S*. *japonicum* infection. We examined p-P65 levels in liver tissues infected with *S*. *japonicum*- and in RAW264.7 cells stimulated SEA. Our findings indicated that deletion or inhibition of MST1 with XUM-XP-1 led to increased levels of p-P65. Furthermore, in line with other recently published studies, we demonstrated that NF-κB was crucial for the expression of inflammatory cytokines in MST1 knockout macrophages, as inhibition of P65 significantly reduced the expression of these cytokines in RAW264.7 cells [39].

Our findings emphasize the role of MST1, but do not rule out the potential involvement of MST2 in analogous biological processes. Both MST1 and MST2 are key kinases in the Hippo signaling pathway, often functioning redundantly in the regulation of processes such as cell growth, apoptosis, and tissue regeneration. Studies have shown that MST2 plays a role in regulating immune responses and influencing macrophage polarization [31]. While our study primarily focused on the impact of MST1 on macrophage polarization and liver fibrosis, it is possible that MST2 may also potentially influence these processes through similar or distinct mechanisms.

In conclusion, our study demonstrates that MST1 plays a protective role in liver fibrosis by upregulating CD36 expression through p-PPARγ at S273, while simultaneously suppressing the inflammatory response by inhibiting p-NF-κB signal in macrophages (S4 Fig. *A Model for MST1-mediated protection in liver fibrosis*). These findings underscore the significance of MST1 in modulating macrophage function during *S*. *japonicum* infection and suggest potential avenues for therapeutic interventions in liver fibrosis.

## Materials and methods

### Ethics statement

All experiments involving mice were performed according to the recommendations of the Laboratory of Animal Welfare and Ethics Committee (LAWEC) of China. The LAWEC Committee of the National Institute of Parasitic Diseases, Chinese Centre for Disease Control and Prevention approved the protocol.

### Antibodies and chemicals

Antibodies used for western blot analysis: phospho-MST1 (Cat# 49332)/MST1 (Cat# 14946), phospho-MOB1A (Cat# 8699)/MOB1A (Cat# 13730), phospho-ERK2 (Cat# 4370)/ERK2 (Cat# 4695), phospho-P38 (Cat# 4001)/P38 (Cat# 8690) were purchased from Cell Signaling Technology (Beverly, MA, USA). phospho-P65 (Cat# 5006)/ NF-κB P65 (Cat# 2006), Anti-GAPDH (Cat# 7021), phospho-PPARγ (S273, Cat# 3675) were obtained from Affinity (San Antonio, USA). Anti-PPARγ (Cat# 7273) and anti-CD36 (Cat# ab133625) were purchased from Santa Cruz (Dallas, USA) and Abcam (Cambridge, UK), respectively.

MST1 kinase inhibitor XMU-XP-1 (SML2233) was purchased from Sigma-Aldrich (St. Louis, MO, USA). The CD36 blocking antibody SR-BI (NB400-104) was purchased from Novus Biologicals (Littleton, Colorado, USA). IKKα inhibitor BAY 11–7082 (ab141228) was purchased from Abcam (Cambridge, UK). Antibodies used for immunofluorescence staining: PE rat anti-F4/80 (Cat#565410, BD pharmingen), rabbit anti-MST1 (Cat# 14946, CST), mouse anti-PPARγ (Cat# 7273, SC.), rabbit anti-CD36 (Cat# ab133625, Abcam), Alexa Fluor 594-goat anti-mouse IgG (H+L)) (ab150116, Abcam), Alexa Fluor 594-goat anti-rabbit IgG (H+L) (ab150080, Abcam), Alexa Fluor 488-goat anti-rabbit IgG (H+L) (ab150077, Abcam).

### SEA preparation

Eggs were obtained from the livers of 40 cercaria-infected mice at 8 weeks of infection. SEA was prepared from homogenized eggs as previously described as previously described in our paper (18) and the protein concentration was determined by BCA Protein Assay Kit (Pierce, Thermo Scientific, USA).

### Cell culture and treatment

The RAW264.7 cells, primary peritoneal macrophages (PEMs) from *Mst1*^*+/+*^ and *Mst1*^*ΔM/ΔM*^ mice were cultured in RPMI1640 media supplemented with 10% fetal bovine serum and penicillin-streptomycin. All the cells were stimulated with or without SEA (50 μg/ml) for 24 hours. For some RAW264.7 cells, MST1 kinase inhibitor XMU-XP-1(3 μm) was added during the last 6 hours of SEA stimulation to detect the nuclear localization of PPARγ, expression of CD36, as well as phosphorylation levels of ERK2, P38, NF-κB and PPARγ. For NF-κB inhibition, cells were pre-treated with IKKα inhibitor (1 μM, BAY 11–7082, Abcam, ab141228) for 1 hours, before SEA and XMU-XP-1 addition.

### Mice, parasites and infection

Myeloid cell (including macrophages)-specific *Mst1*-deficient mice(*Mst1*^*flox/flox*^; *LysM-Cre*, termed *Mst1*^*△M/△M*^)obtained from the Tao lab at Fudan University, Shanghai, China, has been described [14]. *Mst1*^*+/+*^ mice were used as controls in experiments with *Mst1*^*△M/△M*^ mice. Female 6- to 8-week-old C57BL/6 mice were purchased from Model Animal Center of Nanjing University (Nanjing, China). All mice were bred in specific pathogen-free barrier conditions in the Anhui Medical University animal facility.

Mice were infected percutaneously with the *S*. *japonicum* cercariae (Chinese mainland strain) as we previously described [18]. Briefly, female 6 to 8 week-old *Mst1*^*+/+*^ and *Mst1*^*△M/△M*^ mice, were infected with 20 ± 2 cercariae via their abdominal skin. Infected mice were sacrificed at 12 weeks post-infection for samples collection. The protocols for all animal experiments were approved by the Institutional Animal Care and Use Committee at Anhui Medical University.

### Histology and morphological assessment

Formalin-fixed liver tissues from control or *S*. *japonicum*-infected mice were embedded in paraffin using routine procedures. Sections (4 μm) stained with haematoxylin and eosin or Sirius Red were examined under the microscope (Axioskop, Zeiss, Germany) for qualitative and quantitative alterations. The level of granulomatous inflammation was microscopically determined using granulomas area. The level of fibrosis was microscopically determined using collagen deposits area. Three different sections per mouse were analyzed by ImageJ in nine mice per group according to routine methods. The percentage of positive staining was determined by two skilled pathologists in a blind manner through digital morphometry (Image-Pro Plus, RID:SCR_007369).

### Western blotting

Liver tissues or RAW264.7 cells were collected and lysed in RIPA buffer supplemented with complete protease inhibitor (Roche). Protein samples were separated in 10% SDS-denatured polyacrylamide gel and transferred onto PVDF membrane (Beyotime, Shanghai, China). The membranes were blocked with 5% skim milk in TBST at RT for 2 hours, then incubated with the following primary antibodies: phospho-MOB1A (Thr35, 1:1000), anti-MOB1A (1:1000), phospho-MST1 (Thr183,1:1000), anti-MST1 (1:1000), anti-GAPDH (1:5000), phospho-P38(1:2000), phospho-P65 (1:1000), P65 (1:1000), phospho-PPARγ (S273,1:1000), anti-PPARγ (1:50). Next, the membranes were incubated with the HRP-conjugated secondary antibody (Thermo Fisher Scientific) for 2 hours at RT. The protein bands were visualized with ECL HRP chemiluminescent substrate reagent kit (Bridgen). ImageJ software was used for quantitation.

### Liver and spleen index of mice

Mice were euthanized 12 weeks after undergoing infection of cercariae of *S*. *japonicum* and the weight of wet livers and spleens were recorded. Liver or spleen index was calculated by the following formula: Liver or spleen index (%) = weight of liver or spleen (g) /weight of body (g) × 100%.

### RNA-Seq

Peritoneal macrophages (PEMs) from *S*. *japonicum*-infected *Mst1*^*+/+*^ and *Mst1*^*ΔM/ΔM*^ mice at 12 weeks, as well as their respective uninfected control mice (three mice per group) were collected for RNA sequencing. Briefly, RNA preparation, library construction and sequencing on an Illumina Novaseq6000 instrument were performed by the commercial service of Genergy Biotechnology Co. Ltd. (Shanghai, China). The expression of the transcript was calculated by FPKM (Fragments Per Kilobase of exon model per Million mapped reads) using Perl. The DESeq2 method was used to screen for differentially expressed genes (DEGs) between the samples, and hierarchical clustering was performed using Cluster. The expression values were visualized by the R package pheatmap. The DEGs with two-fold changes and an adjusted P values ≤ 0.05 were selected for function and signaling pathway enrichment analysis based on GO and KEGG database. Differentially expressed genes exhibiting two-fold changes and Benjamini and Hochberg-adjusted P values ≤ 0.05 were selected.

### RNA extraction and quantitative real-time PCR

RNA was extracted from cells or tissues with TRIzon Reagent (Thermo Fisher Scientific, USA) following the manufacturer’s instructions and cDNA(2 μg) was synthesized with a Reverse transcription kit (TaKaRa, Japan) and incubated at 42°C for 2 minutes to remove genomic DNA then incubated at 37°C for 15 minutes and 85°C for 5 s. qPCR was performed with 2 μl of cDNA, 10 μl of SYBR Master Mixture (TaKaRa, Japan), and 0.8 μl target gene–specifific primers (Sangon Biotech, China) according to the manufacturer’s instructions. Amplification of β-actin was used as an internal control. The sequences of primers were listed in Tab S2. Gene expression was normalized to β-actin using the 2^-ΔΔCT^ method.

### Phagocytosis assays and CD36 blockade experiment

Phagocytosis assay was assessed using FITC-Dextran 40,000 (Sigma, USA). The purified PEMs from *Mst1*^*+/+*^ mice and *Mst1*^*△M/△M*^ mice were stimulated with SEA (50 μg/ml) for 24 hours. After washing twice with cold PBS, the PEMs were incubated with FITC-Dextran in 2 ml DMEM medium containing 10% FBS at 37°C or 4°C for 1 hour with the ration of cell to FITC-Dextran: 5×10^6^ vs 1 mg. The cells were washed and analyzed by flow cytometry or immunofluorescence. The phagocytic ability of the PEMs is represented by mean fluorescence intensity (MFI), calculated by subtracting the MFI at 4°C from the MFI at 37°C. For immunofluorescence detection, the percentages of FITC^+^ cells in both groups of F4/80-positive cells were calculated.

For the phagocytic function assay with CD36 blockade, WT PEMs were pre-treated with CD36 blocking antibody SR-BI (1:300) for 1 hour before and maintained throughout the 24-h SEA stimulation, and then subjected to FITC-Dextran uptake assay. The phagocytosed cells were used for mRNA detection of macrophage phenotype-related genes.

### Liver mononuclear cells preparation and flow cytometry analysis

Liver granuloma cells were isolated from 12-week infected mice using 0.2% type IV collagenase (Sigma) as previously described with minor modify [18]. Briefly, liver perfusion was performed using PBS through the portal vein followed by cutting of the inferior vena cava. Livers were then cut into small pieces and mashed on 200-gauge steel screens in cold PBS. Contaminating hepatocytes were removed by a brief centrifugation at 60×g for 1 minute. The resulting supernatants were spun in 40% Percoll at 1,260×g for 30 minutes at 25°C to remove all nuclei and cell debris. New pellets containing liver mononuclear cells were treated with Ammonium-Chloride-Potassium (ACK) Lysis Buffer to remove the red cells followed by two washes. When the supernatant is clear, the nonparenchymal cells were counted by Trypan Blue exclusion assay for subsequent experiments.

For surface staining, liver granuloma cells from infected mice and mononuclear cells from control mice were washed with staining buffer. Nonspecific antibody binding was blocked by incubating cells with 10% rat serum for 20 min at 4°C followed by incubation with combinations of primary antibodies for 30 min at 4°C. The following conjugated antibodies were used: CD11B PE (clone M1/70; BD), Ly6C APC (clone HK1.4; Thermo Fisher), F4/80 FITC (cloneBM8; Biolegend), CD36 FITC (clone 72–1; Ebioscience). Cell viability was assessed with DAPI (Sigma) according to manufacturer’s protocols.

For intracellular staining, liver granuloma cells from infected mice and liver mononuclear cells from control mice were stimulated with phorbol 12-myristate 13-acetate (PMA;25ng/ml, Sigma), ionomycin (1μg/ml, Sigma), and GolgiPlug (1μg/ml, BD Pharmingen) for 4 hr. Cells were stained for surface markers, such as CD3 PECy5.5 (17A2,BD) and CD4 FITC (GK1.5, BD, and then fixed, permeabilized and stained for intracellular IL17A PE (clone TC11-18H10, BD), IL4 PE (clone 11B11, BD) or IFNγ PE(clone XMG1.2, BD). Cells were submitted for flow cytomet ry on a FACS Calibur system (BD Biosciences). Data were analyzed using the Flowjo V10 software (FlowJo, RRID: SCR_008520).

### Immunofluorescence staining

For macrophages, RAW264.7 cells or PEMs (3×10^5^) were seeded onto coverslips in 24 well dishes. Cells were fixed with 4% paraformaldehyde for 20 minutes and permeabilized with 0.4% Triton X-100 in PBS for 15 minutes at RT. Unspecific binding sites were blocked with 10% goat serum for 1 hour at RT. Cells were stained with the primary antibodies, mouse PPARγ (Santa Cruz, 1:200) or rabbit anti-CD36 (Abcam, 1:200) followed by Alexa Fluor-594 conjugated-goat anti-mouse IgG (H+L) or Alexa Fluor 488-goat anti-rabbit IgG (H+L) secondary antibody (Abcam, 1:400). This was followed by 3× washes with PBS. The nuclei were visualized using DAPI nucleic acid stain (Solarbio) at 4°C for 30 minutes.

For liver sections, paraffin tissue sections were deparaffinized using routine methods. Antigen retrieval was performed by water bath at 95°C for 15 minutes in 1 mM sodium citrate buffer (pH 8). Non-specific binding was blocked with 5% bovine serum albumin for 1 hour at RT followed by an overnight incubation at 4°Cwith primary antibodies against the antigens in murine livers: rabbit anti-MST1(Cell signaling technology, 1:1000), rabbit anti-CD36 (Abcam, 1:200). Sections were then washed in PBS followed by incubation with Alexa Fluor 488 conjugated- or Alexa Fluor 594 conjugated-goat anti-rabbit IgG (H+L) secondary Abs (Abcam, 1:400). The macrophage marker F4/80 (BD pharmingen, 1:200) was directly detected using PE anti-F4/80, incubated at 4°C for 30 minutes.

Fluorescence images were acquired using a Zeiss LSM 800 confocal fluorescence microscope (Carl Zeiss). Data were analyzed using ImageJ. In Fig 5, we measure the mean fluorescence intensity of CD36 within the co-positive region of F4/80 and CD36 per FOV by ImageJ. Normalized mean fluorescence intensity of CD36 was standardized by the ratio of each mean fluorescence intensity was divided by the maximum fluorescence intensity.

In Fig 7, we calculate the mean fluorescence intensity of CD36 for each field of view, which was obtained by calculating the total fluorescence intensity of a given area within each field of view and dividing it by the size of the given area. Normalized mean fluorescence intensity of CD36 was standardized by the ratio of each mean fluorescence intensity was divided by the maximum fluorescence intensity. Count the number of CD36 positive cells and total cells and proportion of positive cells were calculated using ImageJ. The fluorescence intensity of CD36 overlaid with F4/80+ cells was analyzed by Image J.

### Statistical analysis

Statistical analysis was performed using SPSS software (version 20). Data normality was evaluated with the Shapiro-Wilk test. Student’s *t*-test was used for two-groups comparisons and one-way ANOVA for multiple comparisons, with post-hoc tests applied when necessary. For non-normally distributed data (*P*≤0.05), the Mann-Whitney U test was used for two-group comparisons and the Kruskal-Wallis H test for more than two groups. All data are shown as mean ± SEM. Significance was set at P value <0.05, with levels indicated as: *P<0.05, **P<0.01, **P<0.001.

## Supporting information

S1 Fig No significant differences in the numbers of eggs between Mst1^+/+^ and Mst1^ΔM/ΔM^ mice. Tissue (0.1 g) removed from the left side of the livers of individual animals was digested with 5 ml of 5% KOH at 37°C for 5 h. The eggs in 0.1 ml of the digested tissues were quantified by smear examination under the microscope. Data were expressed as mean ± SEM, n = 9 mice per group, NS = no significant, statistical analysis was performed by Student’s t test.
(TIF)

S2 Fig Gating strategy for liver lymphocyte cytometry data. Dead cells were excluded by DAPI stains. Myeloid cells and lymphocytes were then gated based on forward scatter (FSC) and side scatter (SSC) from live cells. Next, cells expressing CD11b, but lacking F4/80 cells are selected for further analysis of Ly6C expression from the myeloid cell population. Concurrently, CD4^+^ cells are selected to evaluate the expression levels of cytokines IL4, IL17, and IFNγ from the lymphoid fraction. Finally, Ly6C high and Ly6C low populations were analyzed within this population.
(TIF)

S3 Fig The expression of the corresponding genes of Fig 5A in PEMs from uninfected Mst1^+/+^ and Mst1^ΔM/ΔM^ mice. RNA-seq analysis was performed on genes of cytokines, chemokines, phagocytosis-related genes and matrix-degrading enzymes between Mst1^+/+^ and Mst1^ΔM/ΔM^ PEMs (n = 3 per group). *P < 0.05.
(TIF)

S4 Fig A Model for MST1-mediated protection in liver fibrosis caused by *Schistosoma japonicum* infection. SEA activates MST1, upregulating CD36 expression via phosphorylation of PPAR at S273. CD36-mediated phagocytosis increases the expression of fibrolytic genes(*Mmps*, *Arg1*), and promotes the transformation of macrophages into an anti-fibrotic phenotype. Additionally, MST1 negatively regulates the NF-κB-dependent inflammatory genes *(Il1β*, *Il23*, *Il17 and Tnfα*)expression by inhibiting NF-κB phosphorylation induced by SEA stimulation. The shift towards an anti-inflammatory and anti-fibrotic (named Restorative) phenotype results in the amelioration of schistosomiasis-induced liver fibrosis.
(TIF)

S1 Table Source data of S3 Fig, the list of corresponding genes in Fig 5A of PEMs from control Mst1^+/+^ and Mst1^ΔM/ΔM^ mice. The data for S1 Fig obtained from the RNA sequencing data of the peritoneal macrophages (PEM) from MST1^+/+^ and MST1^△M/△M^ mice.
(XLSX)

S2 Table The list of sequences of primers in this study. (XLSX)
