# Supplementary figures and images for: Macrophage MST1 protects against schistosomiasis-induced liver fibrosis by promoting the PPARγ-CD36 pathway and suppressing NF-κB signaling
Source: PLoS Pathog. 2024 Dec 19;20(12):e1012790. doi: 10.1371/journal.ppat.1012790 (PMC11785294; doi:10.1371/journal.ppat.1012790)

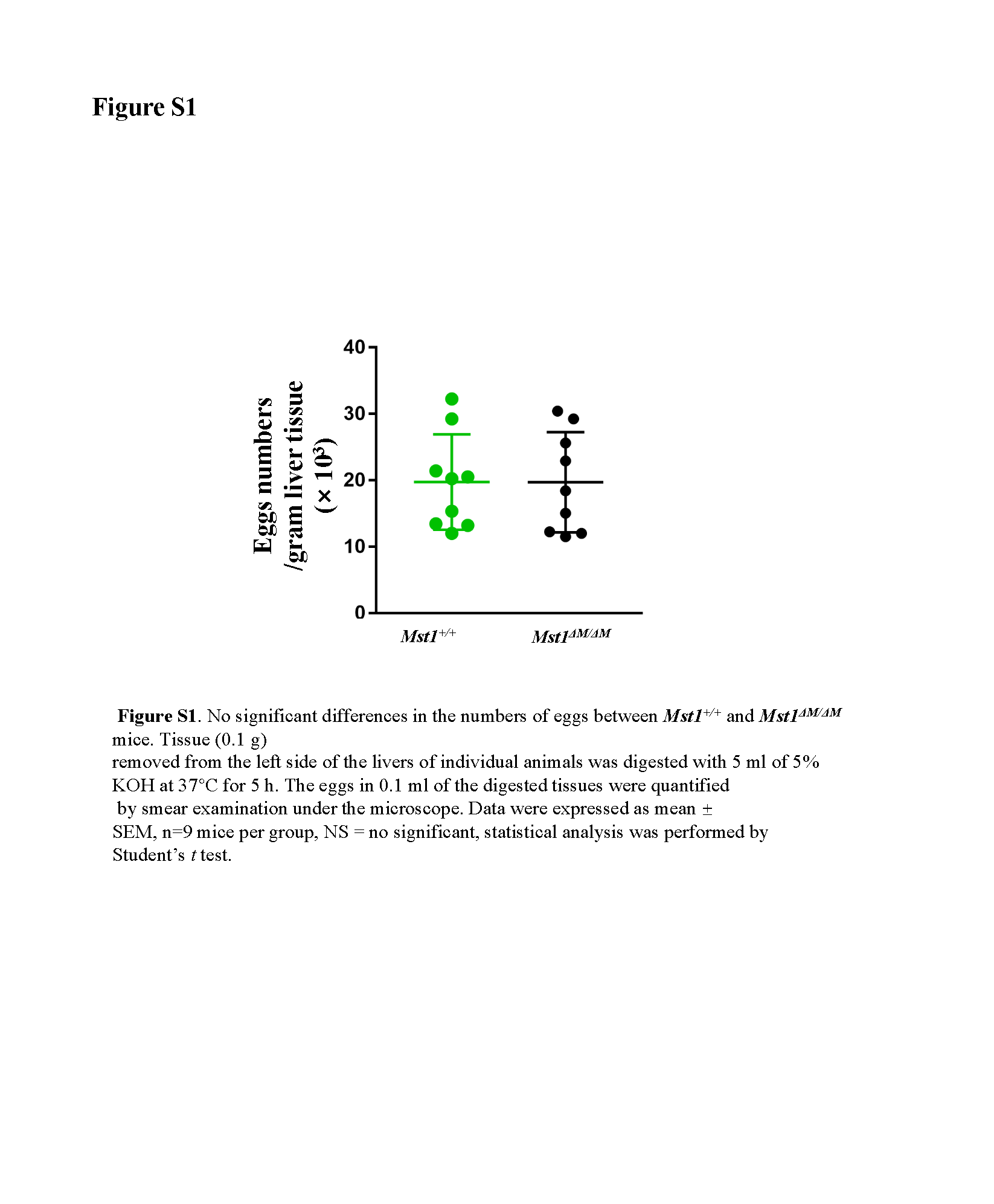

Supplement: S1 Fig — Tissue (0.1 g) removed from the left side of the livers of individual animals was digested with 5 ml of 5% KOH at 37°C for 5 h. The eggs in 0.1 ml of the digested tissues were quantified by smear examination under the microscope. Data were expressed as mean ± SEM, n = 9 mice per group, NS = no significant, statistical analysis was performed by Student’s t test. (TIF) [file ppat.1012790.s001.tif]

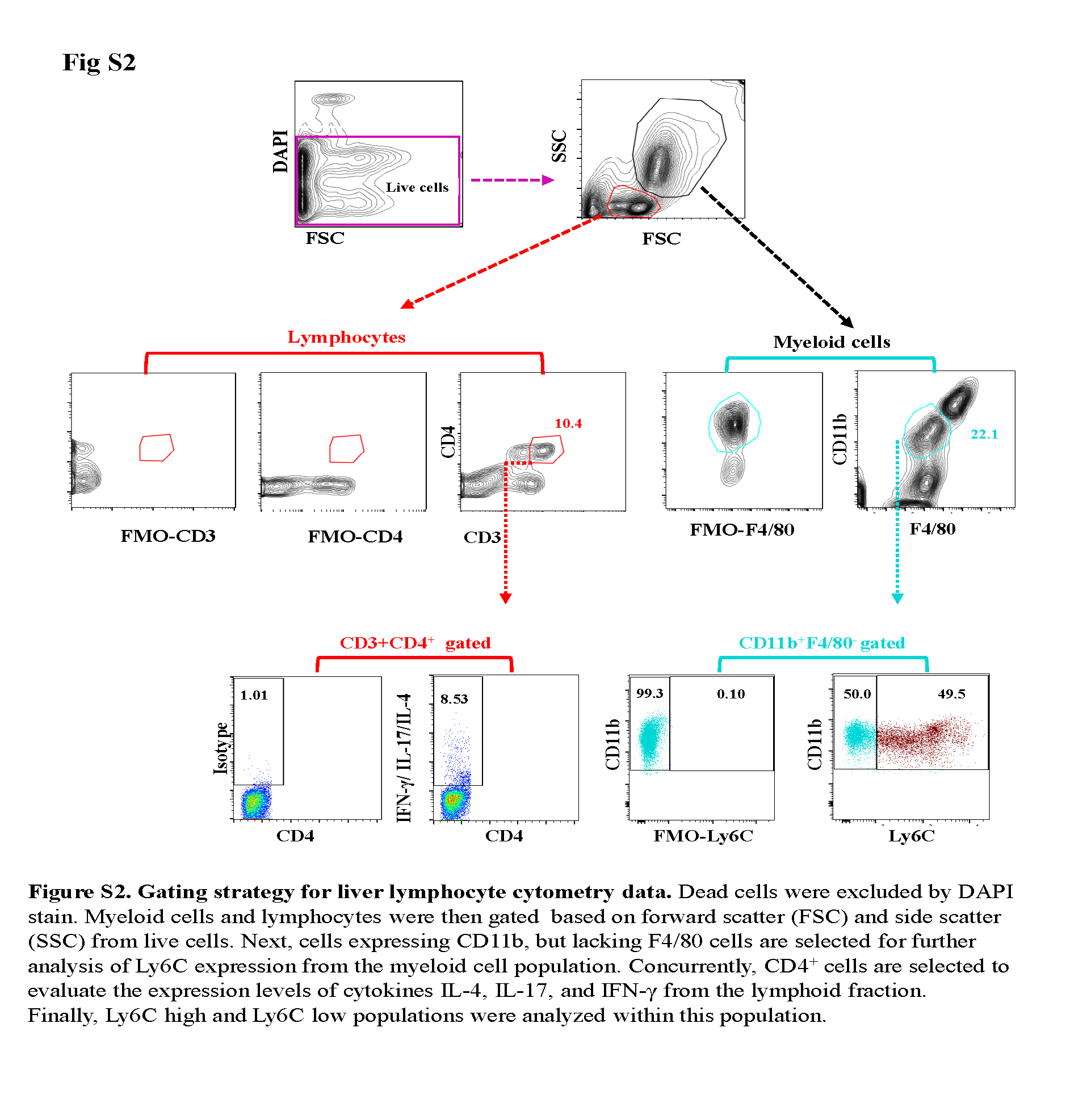

Supplement: S2 Fig — Dead cells were excluded by DAPI stains. Myeloid cells and lymphocytes were then gated based on forward scatter (FSC) and side scatter (SSC) from live cells. Next, cells expressing CD11b, but lacking F4/80 cells are selected for further analysis of Ly6C expression from the myeloid cell population. Concurrently, CD4+ cells are selected to evaluate the expression levels of cytokines IL4, IL17, and IFNγ from the lymphoid fraction. Finally, Ly6C high and Ly6C low populations were analyzed within this population. (TIF) [file ppat.1012790.s002.tif]

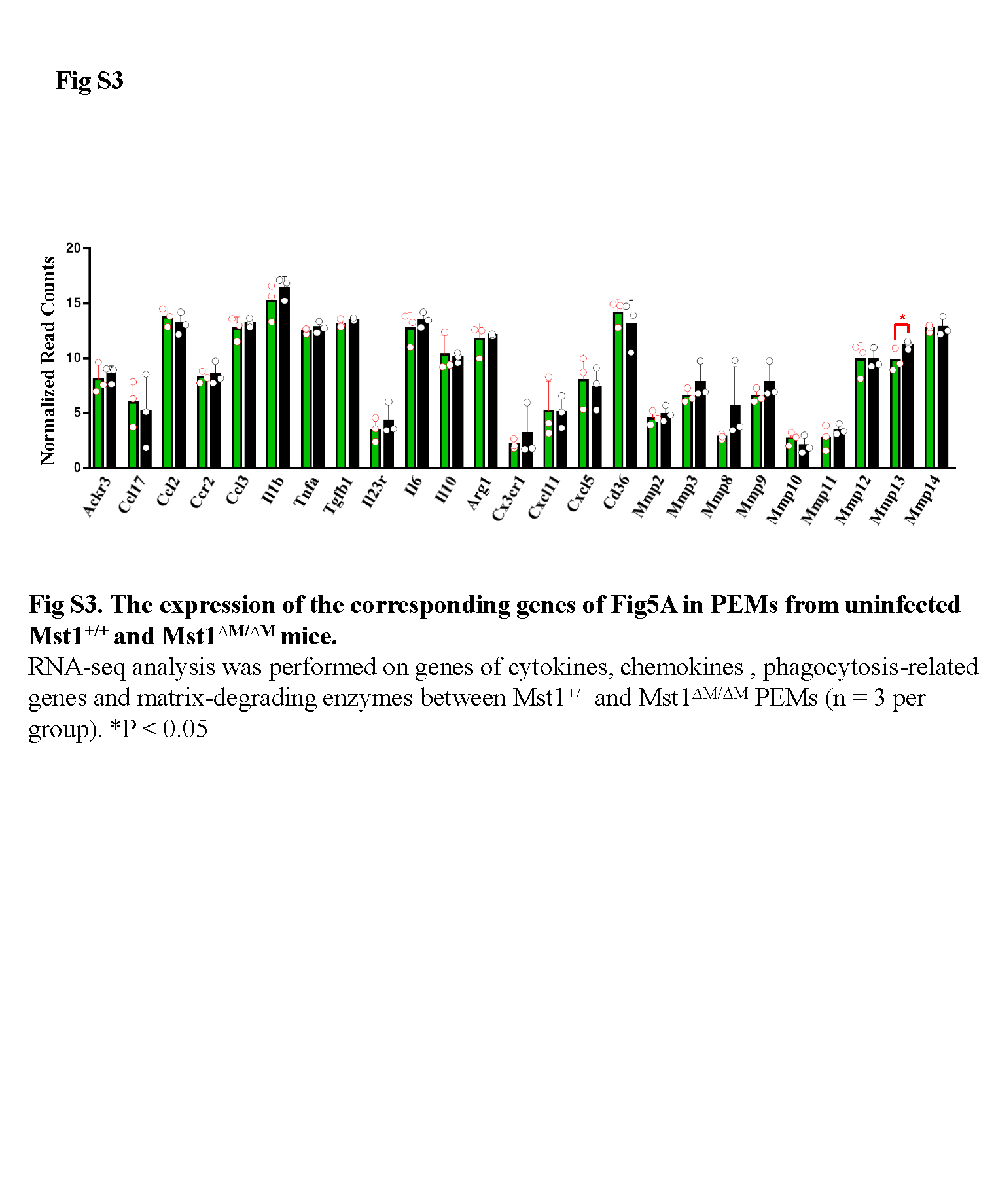

Supplement: S3 Fig — RNA-seq analysis was performed on genes of cytokines, chemokines, phagocytosis-related genes and matrix-degrading enzymes between Mst1+/+ and Mst1ΔM/ΔM PEMs (n = 3 per group). *P < 0.05. (TIF) [file ppat.1012790.s003.tif]

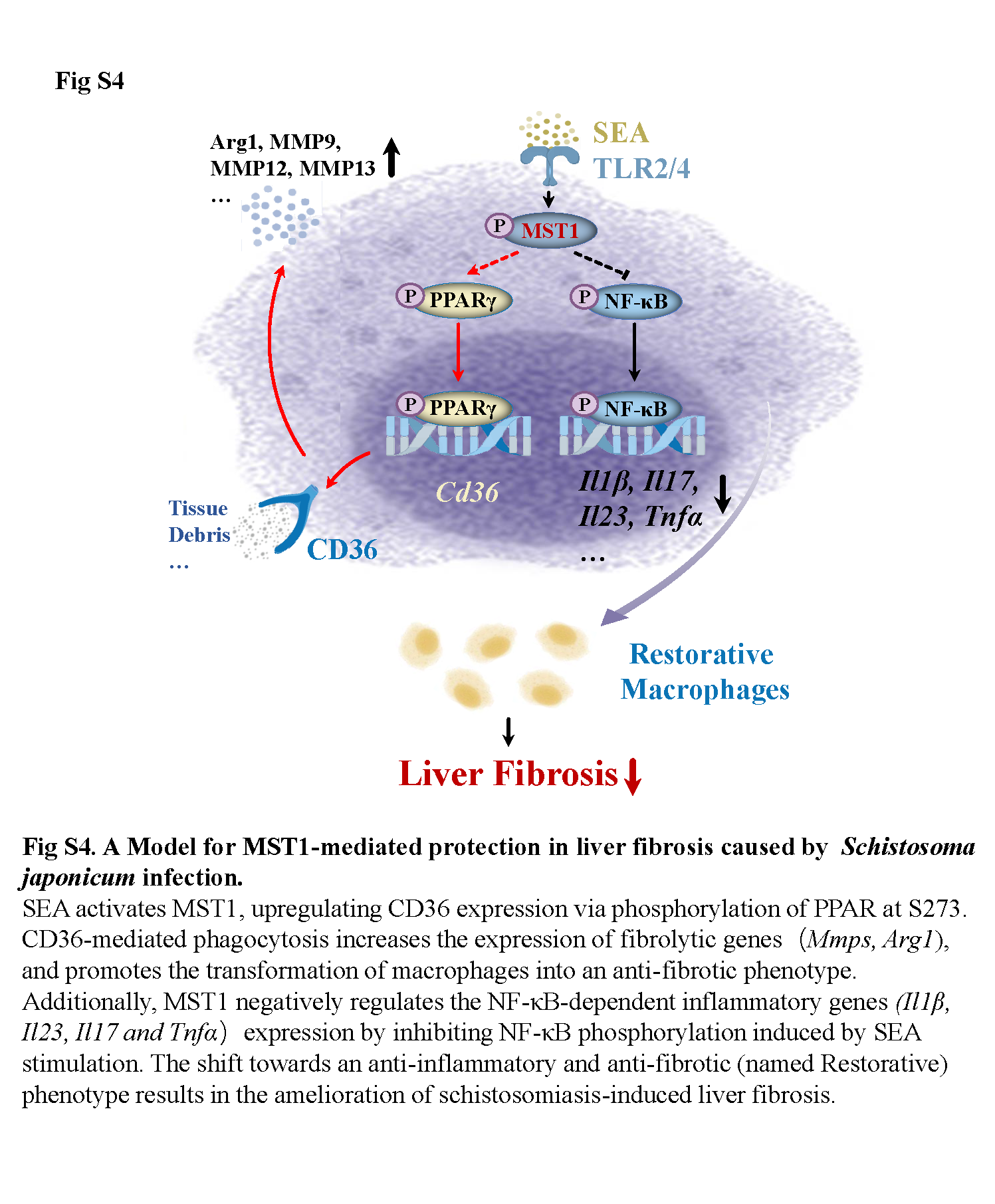

Supplement: S4 Fig — SEA activates MST1, upregulating CD36 expression via phosphorylation of PPAR at S273. CD36-mediated phagocytosis increases the expression of fibrolytic genes(Mmps, Arg1), and promotes the transformation of macrophages into an anti-fibrotic phenotype. Additionally, MST1 negatively regulates the NF-κB-dependent inflammatory genes (Il1β, Il23, Il17 and Tnfα)expression by inhibiting NF-κB phosphorylation induced by SEA stimulation. The shift towards an anti-inflammatory and anti-fibrotic (named Restorative) phenotype results in the amelioration of schistosomiasis-induced liver fibrosis. (TIF) [file ppat.1012790.s004.tif]
